# Comparison of Atrial Fibrillation in the Young versus That in the Elderly: A Review

**DOI:** 10.1155/2013/976976

**Published:** 2013-01-22

**Authors:** Rajiv Sankaranarayanan, Graeme Kirkwood, Katharine Dibb, Clifford J. Garratt

**Affiliations:** ^1^Unit of Cardiac Physiology, Cardiovascular Research Group, 3rd Floor, Core Technology Facility, The University of Manchester, M139PL, Grafton Street, Manchester M13 9NT, UK; ^2^Manchester Heart Centre, Manchester Royal Infirmary, Oxford Road, Manchester M13 9WPL, UK

## Abstract

The incidence and prevalence of atrial fibrillation (AF) are projected to increase significantly worldwide, imposing a significant burden on healthcare resources. The disease itself is extremely heterogeneous in its epidemiology, pathophysiology, and treatment options based on individual patient characteristics. Whilst ageing is well recognised to be an independent risk factor for the development of AF, this condition also affects the young in whom the condition is frequently symptomatic and troublesome. Traditional thinking suggests that the causal factors and pathogenesis of the condition in the young with structurally normal atria but electrophysiological “triggers” in the form of pulmonary vein ectopics leading to lone AF are in stark contrast to that in the elderly who have AF primarily due to an abnormal substrate consisting of fibrosed and dilated atria acting in concert with the pulmonary vein triggers. However, there can be exceptions to this rule as there is increasing evidence of structural and electrophysiological abnormalities in the atrial substrate in young patients with “lone AF,” as well as elderly patients who present with idiopathic AF. These reports seem to be blurring the distinction in the pathophysiology of so-called idiopathic lone AF in the young versus that in the elderly. Moreover with availability of improved and modern investigational and diagnostic techniques, novel causes of AF are being reported thereby seemingly consigning the diagnosis of “lone AF” to a rather mythical existence. We shall also elucidate in this paper the differences seen in the epidemiology, causes, pathogenesis, and clinical features of AF in the young versus that seen in the elderly, thereby requiring clearly defined management strategies to tackle this arrhythmia and its associated consequences.

## 1. Epidemiology

There has been a worldwide increase in the ageing population, and as age is the most significant risk factor for AF, AF cases are predicted to reach nearly 16 million in the USA and 25 to 30 million in Europe by 2050 [1, 2]. The prevalence of AF shows a strong age dependence varying from 0.5% in patients aged <40 years to 5% in patients aged >65 years and nearly 10% amongst octogenarians [3–6]. Both the Framingham Heart Study and the Rotterdam Study estimated that the lifetime risk for development of AF in adults >40 years and at the age of 55 years respectively to be approximately 1 in 4 [7, 8]. The Cardiovascular Health Study which was a large population study of 5201 elderly adults (age ≥ 65 years) showed an incidence of 17.6 and 42.7 events per 1000 person-years amongst men aged 65 to 74 years and 75 to 84 years, respectively, whereas amongst women in the corresponding age groups, the incidences were 10.1 and 21.6 [9]. The SAFE study which was a UK-based multicentre randomised control trial of elderly (≥65 years) patients with AF showed an overall prevalence of 7.2% and 10.3% in those aged 75 years and older, with a 1.6% yearly incidence of new AF [10].

AF in the young in the absence of identifiable causes can be idiopathic or termed as “lone AF,” that is, not associated with comorbidities or obvious cardiac disease. The term “lone AF,” itself was first described nearly sixty years back by Evans and Swann [11]. Traditionally the cut-off age of 60 years has also been included in the diagnosis of lone AF as suggested by ACC/AHA/ESC guidelines [12] and used previously in several landmark epidemiological studies such as the Framingham Heart Study [13] and the Mayo Clinic Study [14]. Old age is indeed one of the strongest risk factors for AF [6] but terming adults over 60 years as “elderly” seems rather controversial in this modern age of improving longevity. The rationale for this “threshold age” therefore has to be questioned [15]. At variance with this age-based definition of lone AF, studies such as that by Kopecky et al. have analysed the outcome of lone AF in patients aged over 61 years, mean age 74 years (range, 61–97 years) [16]. As newer aetiologies are uncovered and the existence of “truly lone AF” becomes increasingly controversial [17–19], the prevalence of   “lone AF” also seems to be steadily “decreasing” in modern studies. A recent study of 3978 AF patients from the Euro Heart Survey by Weijs et al. that excluded age or left atrial size from the definition reported a prevalence of idiopathic AF of 3% out of 3978 patients [20]. The mean age of patients with idiopathic AF in this study was 58 (SD 14) years and nearly half of these (48%) were older than 60 years. Similarly a 30-year follow-up study reported by the Mayo Clinic reported that lone AF constituted only 2% of the total proportion of patients with AF [14]. In contrast, earlier studies showed that lone AF occurs in 1.6% to 30% of all cases of AF, depending on the definition of idiopathic AF or inclusion criteria used [13, 21–23].

## 2. AF Aetiology in the Young 

 Lone AF is a diagnosis of exclusion for which any clinical features of comorbidities or structural cardiac abnormalities that could cause AF must be ruled out. Novel risk factors for AF are increasingly being discovered such as genetic causes, lifestyle factors (such as alcohol consumption, personality traits, and smoking), body mass index, and physical activity, thereby all refuting the diagnosis of “lone AF.” In addition, it can also be argued that occult cardiac pathologies such as hypertension or ischaemic heart disease may well be diagnosed in these patients if they are investigated thoroughly.

### 2.1. Familial

 Nearly seven decades ago, Wolff described a case of familial AF in three brothers [24] and since then studies have found a positive family history of AF in up to a third of AF patients [25, 26]. Having a positive family history especially in younger patients nearly doubles the risk of developing AF [27]. In 1997, Brugada et al. described the first genetic locus on chromosome 10q22-24 in a family with AF segregated as an autosomal dominant trait [28]. In the last few years, genomewide association studies for AF have shown SNPs (small nuclear polymorphisms) at three genetic loci—4q25, 16q22, and 1q21 (reviewed in [29]). Monogenic forms of AF have also been described due to mutations of genes encoding for potassium channels (KCNQI, KCNE2, KCNJ2, and KCNA5), sodium channel gene SCN5A, K(ATP) gene, the ABCC9 gene, and the connexin 40 gene GJA5 [30–36]. Attempts to further unravel the interplay of genomics and AF have shown that the genetic basis of AF is both complex and heterogeneous.

### 2.2. Alcohol

 The “holiday heart syndrome” was described in 1978 by Ettinger to explain the association between supraventricular tachyarrhythmias particularly AF and episodes of increased alcohol consumption during weekends and holiday binge drinking by people without structural heart disease [37]. Overall chronic heavy alcohol consumption (>36 g/day) has been shown to increase risk of AF in several studies including the Framingham cohort (reviewed in [17, 38]). Mechanisms of acute alcohol-induced AF include metabolic acidosis, catecholamine release, and electrolyte disturbances whereas chronic overconsumption leads to myocardial fibrosis, dilatation, and autonomic changes [38]. Binge drinking is particularly prevalent amongst young people and alcohol has been identified to potentiate paroxysmal AF in up to two-thirds of cases [39]. In the elderly, the relationship between AF and alcohol intake is more complex however. The Cardiovascular Health Study showed an inverse association between alcohol consumption and risk of AF in patients over 60 years old, with a 4% lower risk for each additional drink per week [9]. Other studies have also shown a lack of association between risk of developing AF and moderate alcohol consumption [40, 41]. 

### 2.3. Obesity

 A meta-analysis by Wanahita et al looking at 16 studies that enrolled 123249 individuals (mean age 56 ± 2 years) demonstrated a 49% increased risk of developing AF due to obesity (relative risk 1.49, 95% CI 1.36–1.64) [42]. Obesity-associated left ventricular hypertrophy and left atrial dilation are postulated to be important causes which lead to AF [43, 44]. A 3–8% increased risk of developing AF has been associated with each unit increase in body mass index [45, 46]. Obstructive sleep apnoea which is also associated with obesity has been shown to portend risk of AF development in individuals <65 years of age [47]. The association between obesity and lone AF is not robust however [19] and in fact taller and leaner adults are reported to be more prone to develop lone AF [42, 48]. 

### 2.4. Sports and Physical Activity

AF has been recognised to be the commonest cause of palpitations amongst young athletes [49]. Endurance athletes such as marathon runners have been shown to have a greater predisposition to develop AF when compared to nonathletes [48, 50–52]. Karjalainen et al. diagnosed lone AF in 5.3% out of a cohort of 228 male cross-country runners (mean age 47.5 years) [53]. Mont et al. reported a four times higher proportion of sports enthusiasts who had lone AF (aged <65 years) in comparison to controls in a Catalonian population (63% versus 15%) [51]. The same group reported that >1500 lifetime hours is the threshold for increased AF propensity [52]. In more than half of the sportsmen with lone AF, a likely vagal precipitant was identified (postprandial, postexercise or at rest). Interestingly, in the recent GIRAFA study, cumulative work-related moderate-to-heavy physical activity has also been shown to predict risk of developing lone AF in middle aged men aged <65 years [48]. A meta-analysis of six case control studies, including 655 athletes and 895 controls (predominantly men), with a mean age of 51 ± 9 years showed a significantly higher risk of AF in athletes (OR = 5.29; 3.57–7.85; *P* = 0.0001) [54]. Increased left atrial volume was shown to strongly predict AF in athletes [54]. Mechanisms for sports-induced AF postulated include enlarged left atrium and left ventricular mass [17], increased vagal tone leading to bradycardia as well as shorter atrial refractory period [17] and hypovolemia [55].

### 2.5. Cardiac Pathologies

 There are a variety of cardiac pathologies associated with AF in the young. These include hypertrophic cardiomyopathy which confers a four-to sixfold greater risk of AF [56]. Prevalence of AF in these patients is relatively high at about 22% and incidence is 2% annually [56]. Even in this pathology, prevalence increases with age and is seen predominantly in the elderly (>60 years age) [56]. Congenital Heart Disease is another risk factor for AF and with improved surgical outcomes, increasing numbers of infants and children are surviving into adulthood. A large Quebec-based population study of about 38000 adult congenital heart disease patients, with a median age of 42 years, showed a prevalence of atrial arrhythmias of 15.1% (three times greater than that seen in the general population [57]). Wolff-Parkinson-syndrome, myocarditis, pericarditis, and dilated cardiomyopathy are some of the other causes of AF in the young. Valvular heart disease secondary to rheumatic fever is also a significant cause of AF in the young in the developing world. 

### 2.6. Other Risk Factors

Behavioural or emotional triggers such as Type A personality [58], stress [58], anger, and hostility in men [59] have also been shown to predispose to development of AF. Other risk factors associated with AF include increased coffee and nicotine consumption [17, 58, 60]. The association with caffeine intake is debatable however as a canine study showed inverse association between risk of AF and intravenous caffeine and no causative role was found in the Danish diet, cancer, and health study [61, 62]. Smoking has been shown to lead to atrial fibrosis which is well recognised to portend AF [63]. Endocrine causes of AF in the young include hyperthyroidism and phaeochromocytoma.

## 3. AF Aetiology in the Elderly

 As shown in the Framingham Heart Study, AF is usually secondary to a variety of cardiac pathologies (ischaemic heart disease, heart failure, and valvular heart disease) as well as systemic disorders (hypertension, diabetes, hyperthyroidism) [6, 64]. This is particularly true in the elderly who also have an increased predisposition of these conditions. However even accounting for other comorbidities, ageing is the strongest independent risk factor that predisposes to AF [6, 65]. The Cardiovascular Health Study showed that the prevalence of AF was 9.1% in the subgroup with clinical cardiovascular disease, 4.6% in the subgroup with only subclinical cardiovascular disease, and 1.6% in the absence of clinical or sub-clinical cardiovascular disease (i.e., lone AF) [22]. Independent risk factors for AF in the elderly included age, treated systemic hypertension, congestive cardiac failure, valvular heart disease, stroke, enlarged left atria size, mitral or aortic valve dysfunction, echocardiographic features of diastolic dysfunction, and raised serum levels of NT-proBNP [22, 66, 67]. Hyperthyroidism is another cause of atrial fibrillation in the elderly and one study reports an AF incidence of 25% amongst hyperthyroid patients older than 60 years in comparison to 5% in those aged less than 60 years [68].

## 4. Pathophysiology of Lone AF

 AF initiation and maintenance is the result of pulmonary vein repetitive activity (triggers), atrial abnormalities (substrate), and remodelling. Lone AF is likely to be initiated and maintained by the interplay of pulmonary vein ectopics and the posterior wall of the left atrium [69, 70]. Any substrate abnormalities noted have previously been attributed to AF-induced structural remodelling rather than deemed to have a causative role in initiating lone AF. There is increasing evidence however of structural as well as electrophysiological abnormalities in the substrate (i.e., atrium) as well in lone AF. For instance, atrial biopsies in such patients have shown fibrotic changes and even other occult pathologies such as myocarditis [71, 72]. More recently, electrophysiological studies in patients with a history of paroxysmal lone AF remote from the arrhythmia have also shown abnormalities strongly suggestive of dysfunctional atrial conduction involved in AF initiation and progression [73]. These include larger atrial volumes, longer effective refractory period, longer conduction time along linear catheters, longer bi-atrial activation time, slower conduction velocity, larger proportion of fractionated electrograms, longer corrected sinus node recover time, and lower mean atrial voltage [73]. Another study of early onset lone AF patients (age less than forty years old) showed a significant difference in P wave morphology thereby implying abnormal interatrial conduction [74]. These findings seem to implicate a causative role for altered atrial electrophysiology in initiating lone AF. However as AF-induced electrical remodelling is well recognised to start early within a few hours of the onset of AF [75], there remains the possibility that these changes could be secondary to very early remodelling. For instance, previous experiments in a goat model of paroxysmal AF by Garratt et al. showed that it takes between 5 days to 4 weeks to develop changes in atrial substrate which then self-perpetuate AF [76, 77]. A variety of studies have also implicated inflammation in having an aetiological role in the initiation and perpetuation of atrial fibrillation due to evidence of raised inflammatory markers such as interleukin-6 and C-reactive protein in AF patients [78, 79]. In view of the multiple confounding factors and comorbidities associated with inflammation, the jury is still out whether it is a cause or consequence of AF [80]. Structural remodelling as a consequence of AF can cause calcium overload and atrial myocyte apoptosis leading to an inflammatory response [81]. In lone AF patients, there have been contradictory results such as from a large study by Ellinor et al. that showed no difference in high sensitive CRP [82] but others have noted high levels of CRP and high sensitive CRP in lone AF patients [83, 84]. Significantly, however, Aviles et al. included patients with hypertension in their definition of lone AF albeit with overtly structurally normal hearts. This could have been a significant confounding factor as hypertension itself is associated with inflammation [83]. Myocardial perfusion imaging in patients with lone recurrent AF has shown isolated perfusion abnormalities indicative of microvascular dysfunction [85]. Echocardiography has also shown evidence of left ventricular diastolic dysfunction in patients thought to have idiopathic AF [86]. 

## 5. Electrophysiological and Structural Alterations in Aged Atria

### 5.1. Initiation of AF in the Aged Atria

 A number of studies (summarised in Table 1) have attempted to unravel the atrial electrophysiological characteristics of aged atria in relation to AF. Whilst most animal and human studies have shown that aged atria have an increased propensity to develop AF [87–93], some studies in elderly AF patients have yielded conflicting results although these could have been influenced by underlying pathology or treatment in patients [94–96]. Increased atrial ERP has been noted in these studies which could be sufficient to overcome any other arrhythmogenic remodelling. There is therefore a need to understand, in the absence of underlying disease, how AF is initiated and maintained in the aged atria.

### 5.2. Initiation of AF

 Why the aged atria are more susceptible to the development of AF in the absence of other risk factors remains poorly understood. We shall describe below how ageing may increase the propensity of both (i) triggered activity in the form of DADs and (ii) reentrant circuits.

### 5.3. Delayed after Depolarizations (DADs) in the Aged Atria

 Potential contributory factors that may predispose to DAD formation and indeed the increased incidence of DADs have been demonstrated in some models of ageing [90, 93]. Wongcharoen et al. observed DADs of greater amplitude in aged rabbit LA pulmonary vein sleeve tissue sections associated with an increase in NCX protein which would provide an additional means to facilitate triggered activity as more depolarising current would flow during any spontaneous Ca^2+^ release and therefore, all things being equal, a smaller spontaneous release would produce a bigger DAD [90]. Some other studies have also described evidence of atrial calcium mishandling in AF (Table 1) [97]. This has been attributed to protein kinase A-induced hyperphosphorylation leading to dysfunctional ryanodine receptor [98]. While decreased SERCA and increased RyR protein expression give us clues to SR function, it would also be of interest to understand how SR Ca^2+^ content responds to age in the pulmonary vein. One study using human atrial tissue from elderly patients (mean age 68 years) has suggested that hyperphosphorylation of phospholamban could be contributory to leaky ryanodine receptors and thus abnormal calcium handling in chronic AF patients [99]. Clearly there is a pressing need to understand how Ca^2+^ homeostasis is achieved in the aged atria and how it is subsequently remodelled in the aged atria in AF. 

### 5.4. Reentrant Circuits in the Aged Atria

 A reentrant substrate can result from altered functional (electrical) properties or structural changes to the atrium and these are discussed below with reference to ageing.

#### 5.4.1. Effective Refractory Period (ERP)

Whilst conflicting results have been noted when analysing the effects of ageing on effective refractory period in both human [95, 96, 100, 101] as well as animal right atria [92, 102, 103], review of various studies indicates that the right atrial ERP is prolonged with ageing [104]. Inconsistency in the literature may relate to variation in anatomical sites studied [105] and, in terms of patient studies, the effects of underlying disease, arrhythmias, drug treatment, or a general lack of studies including very elderly patients as highlighted by Dun and Boyden [104]. The few studies investigating action potential duration (APD) or ERP in aged left atrium also show conflicting results [106, 107]. Furthermore in rabbit pulmonary vein sleeves and left atrial posterior wall APD was prolonged with age [90, 91]. Electrical characteristics and age-associated remodelling of the atrium appear to be region specific [93] and this may underlie differences between the above studies. Interestingly while the prolonged action potential in aged rabbit left atrial posterior wall may be expected to be anti-arrhythmogenic, this gave rise to an increase in APD dispersion (discussed below) which was suggested to potentiate AF [91].

 Many ion channels have yet to be studied in the aged atria and work mainly performed in the dog has shown that there is no age-related change in sodium current density [108]. However *I*_Ca-L_ is depressed, which would be expected to depress the plateau of the action potential [92], and since repolarising currents activate more strongly at positive potentials, this may slow activation and prolong repolarisation, thus prolonging ERP. Of the repolarising currents only *I*_to_ has been assessed and peak as well as sustained *I*_to_ was increased with age in the right atrium [109] but not the left atrium [104]. Indirect evidence suggests *I*_KACH_ may be enhanced in the right atrium with age [103]; however there are no studies investigating age-associated changes in *I*_K1_, *I*_Kur_, *I*_Kr_ and *I*_Ks_, in either atria.

#### 5.4.2. Conduction Velocity

A number of studies have reported age-associated general or directional conduction slowing and resultant spiral-reentrant waves in the right atria mainly in tissue strips but also *in vivo* in various species [100, 111]. In the aged dog, conduction of normal beats was unaltered but premature impulses were slowed suggesting a certain amount of depolarizing current is needed to overcome conduction discontinuities in age [92]. By calculating conduction in the direction of the wavefront, Kojodjojo et al. have shown in human atria that increasing age is associated with decreased propagation velocity in both atria during sinus rhythm and also during pacing [107]. In this respect the reduction of *I*_Na_ noted in AF [112, 113] is potentially significant as it will slow conduction velocity and reduce the excitation wavelength. Based on a very limited number of studies the effects of ageing on peak *I*_Na_ are inconsistent, showing either no change at low stimulation frequencies but reduced at stimulation frequencies relevant to AF (10 Hz) [108] or a decrease [110]. 

 Changes in connexin expression (especially Cx40 and Cx43) have been noted in AF-related remodelling [114] and are also noted in ageing [115]. A single study has shown an age-associated decrease in connexin 43 in the sinoatrial node but unaltered expression of connexins in the right atria [115]. However a change in the distribution of connexins away from the lateral cell edges to the intercalated discs has also been noted in ageing [116]. This is potentially significant, as it will result in anisotropic propagation of excitation and the formation of reentrant circuits [114, 117]. 

 Increased or heterogeneous fibrosis, often associated with advancing age [92, 111], can disrupt the coupling between individual myocytes and result in non homogenous conduction or conduction slowing which can lead to re-entry (for review see [118]). Recent work has demonstrated increased AF stability in long-term AF in goats due to “microfibrosis” separating myocyte bundles [119]. Conduction abnormalities can occur following redistribution without altered expression [120]. 

#### 5.4.3. Dispersion of Conduction Velocity and ERP

ERP dispersion and conduction heterogeneity correlate with AF inducibility and both have been shown to increase with ageing [91, 93, 105].

In summary, what induces and sustains AF in the young may be different to that in the old. Anyukhovsky et al. showed in old dogs that atrial tissue was depolarised, with longer APD and a slower max upstroke and greater variability in APD. In chronic AF both young and old, the atrial cell membrane was hyperpolarised, with slowed upstroke and decreased APD. But chronic AF led to an increase of APD dispersion in adults and a decrease in old dogs. Thus, AF was sustained in two different substrates: one with short AP duration and with expanded heterogeneity of AP parameters (adult) and one with short AP duration but limited heterogeneity (old). These data also suggest that the increased dispersion in atrial electrophysiology that occurs in adults may be an important additional contributory factor for AF stabilization at this age, while the occurrence of fibrosis and slowed conduction of premature beats that has been demonstrated previously may be more contributory in the old.  Table 2 summarises the atrial electrophysiological differences between the elderly versus the young in relationship to the propensity to develop AF.

Histological changes in healthy elderly patients with AF include increased deposition of collagen, adipose tissue and amyloid, atrophy and vacuolar myocyte degeneration and fibrofatty substitution of the sinoatrial node (reviewed in [118]). Aging-related oxidative damage has been shown to portend atrial fibrillation through mitochondrial bioenergetic dysfunction [121]. Imaging studies have also shown age-related dilatation of the pulmonary veins and the atrium, thereby potentiating pulmonary vein triggers as well as substrate-induced AF maintenance through mechanoelectric feedback [122, 123].

## 6. Clinical Features

The clinical presentation of AF varies significantly depending on age and comorbidities. In the young, the initial presentation is usually with paroxysmal AF [124]; persistent AF under the age of 50 is often associated with identifiable causes like structural heart disease, hyperthyroidism, or alcohol excess. Whilst the incidence of both paroxysmal and persistent AF increases dramatically over the age of 60, there is a disproportionate increase in chronic forms [125], with the result that 80% of newly diagnosed AF in octogenarians is of a persistent or permanent form, even in the absence of structural heart disease [126]. Moreover, advanced age is a risk factor for early recurrence after first AF presentation and of rapid progression from paroxysmal to persistent AF [125, 127]. 

AF is classically associated with “typical” symptoms of irregular palpitations, with or without chest pain, breathlessness, or dizziness. Palpitations are reported in 80% of young patients with paroxysmal AF. [128]. In contrast, less than 10% of AF patients over the age of 80 years have palpitations [129] and up to 40% of elderly hospital inpatients found to have AF are entirely asymptomatic [130]. Whilst atypical chest pain is relatively common in young AF patients, in elderly patients anginal chest pain during AF episodes strongly suggests the presence of significant concurrent coronary disease [131] and might be sufficient to warrant investigation for coronary ischaemia even in the absence of typical symptoms of angina.

 AF in elderly patients is frequently diagnosed coincidentally during general health assessment, hospital admission for nonrelated illnesses, or as a result of its complications [132]. A recent randomized controlled study in primary care suggests that implementing targeted opportunistic screening of over 65-year olds, based on a simple annual pulse assessment, is likely to be cost-effective in improving AF detection [10].

## 7. Management of AF

### 7.1. General Principles

The management of AF is concerned with two main aspects; symptom relief (through rate or rhythm control) and prevention of complications. Although certain complications, such as left ventricular dysfunction, may be reduced by these therapies, the prevention of thromboembolic events requires targeted and appropriate antithrombotic therapy. AF management including stroke prevention is dependent on multiple factors including patient age, comorbidities, and disease profile.

### 7.2. Stroke Risk, Bleeding Complications, and Anticoagulation

AF is associated with a 5-fold increase in the risk of strokes, and strokes due to AF are associated with higher mortality and worse functional outcome [133]. Age has a particularly dramatic impact on the risk of AF-associated stroke: between the ages of 50–59 the average lifetime risk is 5% and 3.9% for men and women, respectively, and this rises exponentially to 22.3% and 23.9% between 80 and 84 [134]. Vitamin K antagonists (usually warfarin) or other oral anticoagulants (see below) reduce the risk of strokes by around 60–70%, albeit at the risk of intracranial haemorrhage and other bleeding-related complications [135, 136]. 

AF in the context of mitral valve stenosis, or a prosthetic mitral valve, is considered to be extremely high risk, and anticoagulation is mandatory in the absence of clear contraindications [136]. For nonvalvular AF, the CHADS2 score was introduced in 2001 as a simple scoring system to assess the stroke risk (see Table 3) [137]. However, the influence of age is underestimated by the CHADS2 system; recent studies have indicated that, amongst moderate risk patients with a CHADS2 score of 1, individuals over the age of 75 with isolated AF are at a higher risk of stroke than are younger patients with a single additional risk factor [138, 139]. Therefore a more comprehensive scoring system called CHA(2)DS(2)VASc has been recommended in recent guidelines (see Table 4) [140]. By recognizing a spectrum of major and minor risk factors that warrant treatment with anticoagulation, this system more accurately identifies individuals at truly low risk and extends the use of anticoagulation into the previous medium risk category. Notably, using the CHA(2)DS(2)VASc, anticoagulation is considered to be potentially beneficial for all patients aged over 65 years, whilst the decision in younger patients depends on additional risk factors. 

Although most AF patients are elderly, until recently these patients have been somewhat underrepresented in clinical trials of anticoagulation. The clinical benefit from anticoagulation not only persists but potentially increases with advanced age; for example, the recent ATRIA study demonstrated that AF patients aged over 75 years benefit most from warfarin treatment in terms of absolute and relative reduction in stroke rate [141]. Despite this clear evidence of benefit, anticoagulation is considerably underutilised in this age group, typically with only one-third of over 85s receiving appropriate therapy despite an absence of definite contraindications [142]. 

In physician studies, the most commonly reported concerns with anticoagulation are perceived bleeding risk and a history of falls [143]. Elderly patients certainly do have an increased risk of bleeding complications [144], and whilst all forms of bleed have a potential associated mortality and morbidity, the main concern is intracranial haemorrhage. Although this complication is relatively rare even in the elderly (<1% per year in the BAFTA trial of patients over 75 years old), nevertheless it constitutes 90% of anticoagulation-related deaths [145]. Careful monitoring and control of anticoagulation can improve this balance; the risk of intracranial haemorrhage is only modestly increased with a therapeutic INR between 2-3 but rises sixfold when the INR rises above 3.5. Conversely, targeting a subtherapeutic INR of <1.8 results in a sevenfold reduction in stroke prevention without a reduction in intracranial haemorrhage risk (thus equating to a loss of clinical effectiveness and worse outcomes). “Real-world” studies of anticoagulation in octogenarians, report that the risk of major bleeding (either fatal or requiring transfusion) is 13.1 per 100 patient-years [146]. Whilst this is higher than the rate of 4.7 per 100 patient years seen in younger patients, the highest risk of bleeding was seen in patients with a high CHADS2 score of ≥3. Thus patients who are at highest risk of bleeding are also those who potentially benefit the most in terms of stroke reduction, and the absolute risk reduction in stroke-related mortality exceeds the risk increase of fatal bleeds. The perceived risk of anticoagulation in patients with a history of falls is probably exaggerated; whilst there is a slight excess of nonintracranial bleeds in these individuals, a meta-analysis has calculated that a fall rate of at least 300 per year would be required to negate the stroke-prevention benefit [147]. 

Recently, guidelines have suggested the use of scoring systems such as HASBLED (see Table 5) in order to better assess bleeding risk prior to commencing anticoagulation. A score of ≥3 is considered to represent high risk of bleeding, and caution and careful monitoring recommended [148]. In view of the complexity of managing anticoagulation in the elderly, there has also been substantial interest in alternatives to oral vitamin K antagonists. However, it is clear that aspirin is significantly less effective than warfarin at preventing strokes, and there appears to be no net benefit over the age of 77 [139]. Recent consensus therefore advises against the use of aspirin for AF thromboprophylaxis [149]. As CHA(2)DS(2)VASc allows identification of patients with an exceedingly low stroke risk, aspirin is now considered to be a nonpreferred alternative for young patients with a single risk factor. 

Recently there has been a surge of new anticoagulant alternatives to warfarin. These do not require regular monitoring of clotting profile. Dabigatran is a newly approved oral direct thrombin inhibitor. The RE-LY trial reported that high dose Dabigatran (150 mg b.d.) was more efficacious than warfarin with a similar risk of bleeding complications (intracranial and extracranial), whilst low dose Dabigatran (110 mg b.d.) was noninferior to warfarin with a reduced risk of bleeding [150]. These effects were consistent amongst young and elderly patient subgroups, although there was a slight excess of gastrointestinal bleeds in elderly patients taking higher dose [151]. This drug could therefore provide either improved stroke prevention when used in high doses in young patients or reduced bleeding risk at low doses in the elderly. Rivaroxaban (a factor Xa inhibitor) is another new anticoagulant. The ROCKET-AF trial reported non-inferiority to warfarin in nonvalvular AF, with no significant increase in major bleeding and a lesser incidence of intracranial and fatal bleeding in comparison to warfarin [152]. Of particular relevance to the elderly, the median age in this trial was 73 years (25% were ≥78 years). Apixaban, another factor Xa inhibitor, was reported last year in the Aristotle trial (median age 70 years) to be more efficacious than warfarin and also caused less bleeding [153].

### 7.3. Rate or Rhythm Control?

 There are two main strategies of management in AF rate control and rhythm control with either pharmacological or nonpharmacological options. 

Pharmacological strategies of rate and rhythm control have been compared in multiple studies. Despite clear theoretical benefits of sinus rhythm in the form of improved atrioventricular dynamics and an improvement in cardiac output, there is no significant benefit of a rhythm control strategy in large randomised population studies. Although there have been few investigations exploring the strategies specifically in different age groups, results of five studies are relevant to a consideration of the elderly and have been included in a meta-analysis [154]; this demonstrates a clear trend towards increased mortality associated with a rhythm control strategy in the elderly. Interestingly, the excess deaths do not appear to relate to proarrhythmic effects or specific side effects of the trial drugs, but rather to other factors such as malignancies or lung disease. This suggests that in elderly frail patients, the use of powerful antiarrhythmic with complex drug interactions may uncover latent comorbidities in a form of nonspecific pharmacotoxicity. 

Thus, current guidelines clearly favour a rate-control strategy in the elderly. Initial therapies might include either beta blockers or calcium channel blockers, with digoxin added as appropriate where a second agent is necessary. Whilst previous guidelines emphasised the importance of tight control of ventricular rate (resting heart rate below 80 bpm), it has recently been demonstrated that equivalent outcomes are achieved with a less stringent target of 115 bpm [155]. In elderly patients, where issues such as coexisting conduction system disease, polypharmacology, and renal impairment are common, a more relaxed approach to rate control is likely to reduce the complexity of management, including the need for pacemaker implantation [156]. 

In patients with drug-refractory symptoms due to permanent AF, pacemaker implantation with atrioventricular node ablation results in symptomatic benefit for up to 83% of patients including those over the age of 70 [157]. Many elderly patients with AF have conduction system disease and pacemaker implantation is often indicated for bradycardic indications; AV node ablation in these patients also allows cessation of rate control medications. Moreover, AV node ablation and cardiac resynchronization therapy should also be appropriate where AF coexists with heart failure, with a recent meta-analysis demonstrating significant mortality benefit over pharmacological rate control [158]. 

Although large clinical studies do not clearly favour either a rate or rhythm control strategy, there are factors that suggest that certain subgroups may benefit from a rhythm control strategy. As older patients with comorbidities demonstrate an excess mortality due to drug-related side effects, so it is arguable that young and otherwise healthy patients should not experience these side effects and therefore experience the full benefits from restoration of sinus rhythm, including improved ventricular function, quality of life, and reduced progression to permanent AF. There is some evidence from studies focusing on younger patients suggesting this to be the case, and a convincing case that where a rhythm control strategy is effective, it is associated with reduced symptoms and improved mortality compared with rate control [159], although the data as yet do not suggest that anticoagulation can safely be withdrawn. 

 Factors associated with rapid progression of AF (advanced age and structural heart disease) are also associated with a poor response to rhythm control strategy. Recently, the HATCH scoring system (hypertension, age older than 75 years, previous transient ischemic attack or stroke, chronic obstructive pulmonary disease, and heart failure) has been proposed to identify patients at risk of progression to persistent AF [127]; a HATCH score of ≥2 was associated with a high risk of disease progression despite antiarrhythmic therapy. 

Where a rhythm control strategy is considered appropriate, treatment choices are guided by side effects and contraindications rather than expected efficacy. Where there is no underlying structural heart disease, appropriate first line options include dronedarone, flecainide, propafenone, and sotalol [160]. Although amiodarone is a more efficacious antiarrhythmic [161], it is associated with significant pulmonary, liver and thyroid toxicity with a cumulative and dose-dependent risk; its long-term use in the young therefore requires very cautious consideration.

Dronedarone has recently emerged as an anti-arrhythmic therapy for paroxysmal AF. It appears to be somewhat less efficacious than amiodarone, but associated with less side effects [162]. The recent ATHENA [163] study suggested that dronedarone may impart a benefit in terms of reduced mortality and hospital admission compared to placebo in certain high risk patient groups, but data showing benefit over other anti-arrhythmic therapies are lacking. Importantly, it is also contra-indicated in patients with permanent AF or decompensated heart failure.

In recent years, the major advance in rhythm management has been in nonpharmacological therapy, namely, left atrial ablation. Since the first report by Haissaguerre et al. that pulmonary vein isolation reduced AF recurrence by eliminating spontaneous focal discharges from pulmonary veins that initiated AF, there have been significant advances in ablation techniques [70]. This treatment strategy is particularly efficacious in patients with lone AF and thus the European Society of Cardiology guidelines recommend left atrial ablation (Class IIa recommendation) for symptomatic patients with paroxysmal or persistent AF who have failed to respond to trial of anti-arrhythmic medication [160]. Whilst complications are reported in up to 5% of cases, multiple studies have demonstrated freedom from AF leading to significant improvements in symptoms and quality of life [164, 165]. Results in paroxysmal AF demonstrate that pulmonary vein isolation gives satisfactory symptom control and freedom from AF in around 70 to 80% patients; however, persistent AF generally requires more complicated procedures involving multiple lesions throughout the left atrium (success rates of 65–75%) [166]. Several studies have also demonstrated that catheter ablation is more effective than anti-arrhythmic therapy [167–169] at controlling symptoms as well as restoring sinus rhythm, although there is no firm evidence of benefit in terms of mortality or stroke prevention. Most studies have included patients only under the age of 65, although there have been some small studies suggesting that the procedures can be performed safely in older patients with structurally normal hearts [170]. 

## 8. Conclusion

 To summarise, AF is a heterogeneous condition, with significant differences in its epidemiology, pathogenesis, clinical presentation and management across age groups (shown in Table 6). Older patients are more likely to have an abnormal substrate and present at an advanced stage with atypical symptoms and associated comorbidities. Whilst there have been a few reports of substrate abnormalities in young patients with idiopathic AF thereby implying a causative role, there is not yet conclusive evidence that these do not simply represent AF-induced atrial remodeling. With increasing recognition of rare aetiologies of what was previously deemed to be “idiopathic AF” and the archaic cut-off age to define “lone AF,” the term “lone AF” itself is becoming increasingly obsolete. It is all the more pressing therefore to exclude any occult risk factors for AF as this could influence prognosis and management. The important differences between AF in the young and that in the elderly necessitate clearly defined diagnostic and targeted management strategies to relieve symptoms as well as to prevent complications.

## Figures and Tables

**Table 1:** Studies investigating relationship between atrial electrophysiological changes and ageing.

Authors	Species	Characteristics	Key findings
Brembilla-Perrot et al. [96]	Human	Patients aged >70 years versus younger	Decreased AF inducibility due to increased atrial ERP
Centurión et al. [89]	Human	Patients with paroxysmal AF during sinus rhythm aged >60 years versus younger	Greater mean number of abnormal right atrial electrograms defined as ≥100 msec duration and, or showing eight fragmented deflections
Roberts-Thomson et al. [87]	Human	Patients aged >60 years versus younger	Greater number of complex fractionated electrograms
Sakabe et al. [94]	Human	Patients without a history of AF or structural heart disease	No relationship between age and inducibility of AF

Calcium mishandling			

El-Armouche et al. [99]	Human	Western blotting used to assess phosphorylation levels of Ca handling proteins in right atrial appendage	Hyperphosphorylation of phospholamban could be contributory to leaky ryanodine receptors and thus abnormal calcium handling in chronic AF patients
Hove-Madsen et al. [97]	Human	Age > 66 years	Higher calcium spark frequency and higher incidence of spontaneous calcium waves in comparison to patients with sinus rhythm
Ono et al. [88]	Rats	Old versus young rats	Glycolytic inhibition has been shown to result in spontaneous AF due to calcium mishandling and early after depolarisation-induced triggered activity
Wongcharoen et al. [90]	Rabbits	Responses of pulmonary vein tissues to rapamycin, FK-506, and ouabain in young and aged rabbits	Increased pulmonary vein arrhythmogenesis secondary to ryanodine receptor dysfunction-resultant calcium mis-handling

Atrial ERP			

Kistler et al. [100]	Human	Electrophysiological and electroanatomical studies in 3 age groups (≥60 years, 31–59 years, and ≤30 years)	Age-associated electrical and structural remodeling (regional conduction slowing, increase in atrial ERP, impaired sinus node function, conduction delay at crista terminalis, and areas of low voltage)
Brembilla-Perot et al. [96]	Human	734 patients (age 16–85 years, mean 61 ± 15 years)	Increased atrial ERP and age >70 years independently predicted reduced AF inducibility
Brorson and Olsson [101]	Human	Right atrial monophasic action potentials recorded in 40 healthy males	No age correlation
Anyukhovsky et al. [92]	Dogs	Young versus old canine atrial	Age-related differences in action potential contour, decreased *I *_CaL_, and slower conduction of early premature beats
Huang et al. [106]	Rats	Adult, middle aged versus aged rats	Age-associated prolongation of the monophasic action potential (mAP) and ERP in the right atrium, but a decrease in mAP and ERP in the left atrium, suggesting a potential reentrant mechanism for AF
Kojodjojo et al. [107]	Humans	Most study subjects suffered from atrioventricular reentrant arrhythmias, syncope, or palpitations and hence these atria were not “healthy”	No change in left atrial ERP with ageing
Michelucci et al. [105]	Humans	17 normal subjects (age range 17–78 years)	Age-related increase in right atrial ERP
Su et al. [103]	Rats	Adults versus aged rats	In response to muscarinic stimulation, ageing-related prolongation of atrial maximum diastolic potential but not of APD
Toda [102]	Rabbit	Rabbit ages varied from 2–360 days old	Age-related prolongation of APD

Ion channel remodelling in ageing and AF			

*I*_ CaL_ Anyukhovsky et al. [92]	Canine atria		Reduced *I*_CaL_
*I*_ Na_ Baba et al. [108]	Canine atria		(i) Peak current unchanged at low stimulation frequencies but reduced at stimulation frequencies relevant to AF
Wu et al. [110]	Rabbit atria		(ii) Decreased in hyperlipidemic aged rabbits
*I*_ to_ Dun et al. [109]	Canine atrium		Increased in the left atrium
*I*_ KAch_ Su et al. [103]	Rat		Indirect evidence of increase [104]

**Table 2:** Electrophysiological differences between the elderly and young that can predispose to AF (summarised from human and animal studies in Table 1).

Features	Elderly	Young
**Impulse initiation**		
(i) Sinus node function	Impaired (leading to longer sinus node recovery times), contributing to abnormal impulse initiation	Generally preserved
(ii) Pulmonary vein ectopic activity	Also contributes to AF pathogenesis although substrate abnormalities have a dominant role in initiation and maintenance	Predominant trigger for AF initiation

**Impulse conduction**		
(i) P wave morphology and duration (usually signifying interatrial conduction)	Abnormal P wave morphology and prolonged interatrial conduction	Usually normal
(ii) Wavefront propagation	Abnormalities noted such as conduction slowing (particularly of premature impulses) thereby contributing to reentrant waves	Usually normal

**Substrate abnormalities**		
(i) Complex fractionated atrial electrograms	Greater number	Lesser than in elderly
(ii) Atrial refractoriness—effective Refractory Period (ERP)	ERP prolonged in the right atrium and could contribute to dispersion in refractoriness	Usually not prolonged
(iii) Action potential duration (APD)	Prolonged in the right atrium	Generally within normal limits
(iv) Regional atrial voltage differences	Larger atrial volumes with more number of low voltage areas	Atria usually of normal size and mean voltage within normal limits

**Table 3:** CHADS2 scoring system [137].

	Comorbidity	Score
C	Congestive heart failure	1
H	Hypertension	1
A	Age ≥ 75 years	1
D	Diabetes mellitus	1
S2	Stroke or TIA	2

CHADS2 score 0; annual stroke risk 1.9%, >1; annual stroke risk 2.8 = 18.2%.

**Table 4:** CHADS2VAS2C scoring system [140].

	Comorbidity	Points
C	Congestive heart failure (or left ventricular systolic dysfunction)	1
H	Hypertension: blood pressure consistently above 140/90 mmHg (or treated hypertension on medication)	1
A2	Age ≥ 75 years	2
D	Diabetes mellitus	1
S2	Prior stroke or TIA or thromboembolism	2
V	Vascular disease (e.g., peripheral artery disease, myocardial infarction, aortic plaque)	1
A	Age 65–74 years	1
Sc	Sex category (i.e., female gender)	1

**Table 5:** HASBLED scoring system [148].

Hypertension (BP > 160 without control)	1
Renal disease (dialysis, transplant, Cr > 2.6 mg/dL or >200 *μ*mol/L)	1
Liver disease (cirrhosis, bilirubin > 2x normal, AST/ALT/AP > 3x normal)	1
Stroke	1
Predisposition to bleeding or previous major bleed	1
Labile INR (unstable/high INRs, <60% time in therapeutic range)	1
Age ≥ 65	1
Medications (antiplatelets or NSAIDS)	1
Alcohol excess	1

Risk of spontaneous major bleeding (episodes per 100 patient years)	

Score 0-1	1.02–1.13
Score 2-3	1.88–3.74
Score ≥ 4	≥8.7

**Table 6:** Common differences between AF in the young versus elderly.

	AF in the young	AF in elderly patients
Causes	(i) Idiopathic	(i) Ischaemic heart disease
	(ii) Genetic	(ii) Heart failure
	(iii) Alcohol, smoking	(iii) Valvular heart disease
	(iv) Personality traits	(iv) Hypertension
	(v) Body mass index	(v) Cardiomyopathies
	(vi) Endurance sports	(vi) Hyperthyroidism
	(vii) Cardiac pathologies	(vii) Secondary causes such as post operative, infection, pulmonary embolism
	(viii) Endocrine disorders	(viii) Idiopathic
Pathogenesis	Triggers/pulmonary vein Repetitive activity +++ Substrate/atrial abnormalities +	Pulmonary vein repetitive activity ++ Atrial Abnormalities +++
Clinical features	Usually typical symptoms	Atypical symptoms or asymptomatic
Management	Rhythm control preferred Thromboprophylaxis usually not required unless based on CHADS2VASC	Rate control preferred Thromboprophylaxis usually required unless contraindicated

## Abbreviations

AF: Atrial fibrillation
APD: Action potential duration
ERP: Effective refractory period.
